# Impact of financial incentives on alcohol intervention delivery in primary care: a mixed-methods study

**DOI:** 10.1186/s12875-016-0561-5

**Published:** 2016-11-25

**Authors:** Amy O’Donnell, Catherine Haighton, David Chappel, Colin Shevills, Eileen Kaner

**Affiliations:** 1 Institute of Health and Society, Newcastle University, Baddiley-Clark Building, Richardson Road, Newcastle upon Tyne, NE2 4AX UK; 2 Department of Public Health and Wellbeing, Faculty of Health and Life Sciences, Northumbria University, Newcastle upon Tyne, UK; 3 Public Health England, Durham, UK; 4 Balance: The North East Alcohol Office, Durham, UK

**Keywords:** Alcohol drinking, Screening, Brief intervention, Pay for performance, Primary health care, Mixed methods

## Abstract

**Background:**

Local and national financial incentives were introduced in England between 2008 and 2015 to encourage screening and brief alcohol intervention delivery in primary care. We used routine Read Code data and interviews with General Practitioners (GPs) to assess their impact.

**Methods:**

A sequential explanatory mixed-methods study was conducted in 16 general practices representing 106,700 patients and 99 GPs across two areas in Northern England. Data were extracted on screening and brief alcohol intervention delivery for 2010-11 and rates were calculated by practice incentive status. Semi-structured interviews with 14 GPs explored which factors influence intervention delivery and recording in routine consultations.

**Results:**

Screening and brief alcohol intervention rates were higher in financially incentivised compared to non-incentivised practices. However absolute rates were low across all practices. Rates of short screening test administration ranged from 0.05% (95% CI: 0.03-0.08) in non-incentivised practices to 3.92% (95% CI: 3.70-4.14) in nationally incentivised practices. For the full AUDIT, rates were also highest in nationally incentivised practices (3.68%, 95% CI: 3.47-3.90) and lowest in non-incentivised practices (0.17%, 95% CI: 0.13-0.22). Delivery of alcohol interventions was highest in practices signed up to the national incentive scheme (9.23%, 95% CI: 8.91-9.57) and lowest in non-incentivised practices (4.73%, 95% CI: 4.50-4.96). GP Interviews highlighted a range of influences on alcohol intervention delivery and subsequent recording including: the hierarchy of different financial incentive schemes; mixed belief in the efficacy of alcohol interventions; the difficulty of codifying complex conditions; and GPs’ beliefs about patient-centred practice.

**Conclusions:**

Financial incentives have had some success in encouraging screening and brief alcohol interventions in England, but levels of recorded activity remain low. To improve performance, future policies must prioritise alcohol prevention work within the quality and outcomes framework, and address the values, attitudes and beliefs that shape how GPs’ provide care.

**Electronic supplementary material:**

The online version of this article (doi:10.1186/s12875-016-0561-5) contains supplementary material, which is available to authorized users.

## Background

Heavy drinking affects one in four adult primary care patients in the United Kingdom (UK), [1] contributes to 61 disease conditions, [2] and results in a £3.5 billion annual cost to the National Health Service (NHS). [3] Yet it is one of the most important modifiable causes of premature morbidity and mortality. [4] There is strong evidence of effectiveness supporting screening and brief alcohol interventions at reducing consumption, [5] but historically there have been low rates of delivery. [6] Voluntary financial incentives were employed between 1^st^ April 2008 and 31^st^ March 2015 to encourage English general practitioners (GPs) to screen their patients for heavy drinking using a validated self-report questionnaire [7], and deliver brief behavioural interventions to those in need of support. [8]

Under the national alcohol-related risk reduction directed enhanced service (DES) scheme, participating practices were paid £2.38 for each newly registered adult patient recorded as being screened for heavy drinking. [9] Whilst the scheme did not directly remunerate intervention delivery, contractual and audit guidance made clear that those patients identified as drinking at hazardous or harmful levels should be offered a brief or extended intervention. Locally-negotiated enhanced service (LES) schemes for alcohol were also introduced in certain areas. LES schemes varied in their scope and reimbursement rates, although generally they were more generous than their national counterparts and involved a more opportunistic approach to screening. For example, in one area of Northern England, practices received £8.00 for each registered patient aged 16+ (excluding newly registered patients covered via the DES) who screened positive for risky drinking and received brief advice. Both national and local schemes were voluntary, and separate to the Quality and Outcomes Framework (QOF), the principal UK incentive scheme, which links up to 25% [10] of GPs’ income to performance against a series of clinical and organisational priority areas [11]. Currently, the QOF does not include specific payments for alcohol interventions, although GPs are expected to record alcohol consumption in some disease management areas such as hypertension, coronary heart disease and mental health.

Financial incentives have been used to stimulate improvements in healthcare quality in the UK and other parts of the world. [12] Yet the extent to which this approach has been effective remains contested, [13] with concerns voiced over the perverse and unintended consequences of structuring a health system around targets and process. [14–16] In theory, routine data provide a timely, cost-effective and comprehensive information source to support evaluation of incentive schemes. [17] However, evidence highlights their low sensitivity in capturing the management and treatment of complex and chronic conditions. [18] Moreover, even where such data suggest improvements in health-related outcomes, a common criticism of pay-for-performance programmes is that they merely promote better recording of care rather than better care itself. [19] At the extreme end of the scale, there have also been accusations of GPs ‘gaming’ the system, by manipulating recording of care to boost financial reward. [20]

There has been limited research into the impact of financial incentives on alcohol prevention work in England. [21] The primary aim of our study was to assess the impact of two specific national and local pay-for-performance schemes to encourage screening and brief alcohol intervention delivery using routinely recorded Read Code data. Given the use of routine data as a proxy measure of care under such incentive schemes, our secondary aim was to examine the value of such data from the GP perspective.

## Method

A sequential, mixed-methods study was conducted between February 2010 and May 2013. First, we sought to assess the impact of financial incentives on screening and brief alcohol interventions by comparing recorded rates of delivery between those practices receiving financial incentives for alcohol screening and those not receiving additional payments. Second, we used semi-structured interviews to explore GPs’ perceptions of factors influencing the delivery and recording of alcohol interventions in routine consultations.

### Quantitative analyses

#### Data and measures

Read Code data were extracted from a sample of 16 general practices based in Northern England. Read Codes are a standard clinical vocabulary which support encoding of multiple patient events in the UK, including: demographic details; clinical symptoms and diagnoses; and laboratory tests and results. [22] Whilst their principal role is to support clinical practice, Read Codes are also used to evidence the extent to which GPs have met their administrative, legal, and contractual obligations, including those required by financial incentive schemes. [23]

Read Code search strategies were developed for EMIS [24] and SystmOne [25] clinical computing systems, drawing on guidance on the recording of incentivised screening and brief alcohol intervention activity. [26] The key coding outcomes of interest were: [1] administration of an alcohol screening test (four item FAST [27]; three item AUDIT-C [28]; ten item AUDIT questionnaire [7]); and [2] delivery of either brief alcohol advice (sometimes coded as a brief intervention), or an extended brief intervention (which could include additional sessions or longer input). [29] Given the discrete populations covered by each enhanced service scheme (only patients registered during the previous 12 month period were eligible for DES payments, whereas the LES explicitly excluded this group), we extracted aggregated counts of each coding outcome of interest from the total eligible adult population as opposed to newly registered patients only. This approach also served to protect patient confidentiality in smaller practices. Full details of the search strategies are available from the authors.

#### Data and sample

Stratified purposive sampling was used to identify practices based on three variables: NHS area (either area A or B); incentive status (DES only; DES + LES; none); and practice size. In addition to allowing the comparison of incentivised versus non-incentivised delivery rates, this allowed us to compare recorded activity in an area in which a LES had been launched in addition to the DES (area B); and one where only the DES was available (area A). Practice managers were emailed an invitation to participate in the study, which explained the purpose of the research, and what their involvement would entail. A confidentiality agreement was signed between participating practices and the research team. Recruitment took place between March 2011 and April 2013.

#### Analysis

Screening and brief alcohol intervention rates were calculated by dividing the aggregated Read Code counts by the total eligible registered adult patient population for each practice. As indicated above, we used the total adult population as opposed to newly registered patients only as our denominator when extracting data and conducting primary analyses. Taking into account the targeted remit of the DES scheme however, we also estimated the rate of short alcohol screening test administration amongst newly registered patients only. Rates for each coding outcome of interest were grouped by incentive status (DES only; DES + LES; none). 95% confidence intervals were determined with the binomial distribution and calculated using the Wilson Score method [30].

### Qualitative analyses

#### Data and sample

Next, we interviewed 14 GPs to explore their views on delivering and recording screening and alcohol intervention activity. A purposeful sampling strategy was employed to identify a maximum variety of participants based on four criteria: NHS area; incentive status; gender; and employment (salaried or practice partner). We interviewed GPs from the quantitative sample practices above, and additional participants whose practice data had not been assessed, in case the former scrutiny affected their responses. [31] GPs were contacted via telephone or email, at which point the confidential and anonymous nature of the study was emphasised, and written informed consent obtained. Interviews took place at a time and location convenient to the participant, generally at their practice. A topic guide was used to focus discussions on experiences of delivering and recording alcohol-related care (see Additional file 1), however emergent issues were also pursued. Interviews lasted 22-47 minutes (mean = 32 minutes) and were conducted until data saturation occurred.

#### Analysis

Interviews were audio-recorded, transcribed verbatim and anonymised. Analysis was guided using the Framework approach, [32] whereby data were sifted, coded and charted against a coding framework in accordance with initial and emergent themes. NVivo Qualitative Research software (version 9.2) was used for data management and analysis. [33] All interviews were conducted and analysed initially by AO, with emergent themes discussed and refined within the wider research team.

### Mixed methods synthesis

Mixed methods data integration took place at two levels. Results from the quantitative phase informed the content and direction of the interviews with GPs, as well as the identification of interviewees. Findings from both research phases were then drawn together to determine common themes across the study as a whole. As part of this process, a mixed-methods matrix was employed to support cross-case analysis of results from nine practices where both qualitative and quantitative data were available. [34] This allowed the identification of overarching themes as well as helping to highlight areas of divergence and discrepancy.

## Results

### Quantitative results

Data were collected in 16 practices, representing 106,700 patients and 99 GPs in Northern England. Five practices were signed up to the DES, seven to the DES + LES, and four had no incentive scheme. Areas A and B were located adjacently within the same region and were broadly similar in terms of demography and deprivation. The key difference between the two areas concerned the additional local incentive (LES) available to practices in area B. As take-up of both the LES and the national DES achieved saturation during 2010-11 in area B, we were unable to recruit non-incentivised practices to the study from that part of the region. Additional practice characteristics are detailed in Table 1, with all data summarised in Table 2.

**Table 1:** Key characteristics of sample practices

		Enhanced Service for Alcohol Status			Size of Practice		Index of Multiple Deprivation
	ID	No Enhanced Service	DES only	DES + LES	Number of registered patients*	No. GPs	Quintile (1^st^ = most)
Area A	P01	1	0	0	1,400	5	1^st^
	P02	1	0	0	16,500	13	2^nd^
	P03	1	0	0	7,600	8	4^th^
	P04	1	0	0	8,000	6	1^st^
	P05	0	1	0	9,800	10	3^rd^
	P06	0	1	0	3,000	2	2^nd^
	P07	0	1	0	6,800	7	3^rd^
	P08	0	1	0	600	3	1^st^
	P09	0	1	0	8,800	7	4^th^
Area B	P10	0	0	1	4,900	4	1^st^
	P11	0	0	1	1,200	4	1^st^
	P12	0	0	1	3,100	4	1^st^
	P13	0	0	1	4,800	4	1^st^
	P14	0	0	1	6,200	5	2^nd^
	P15	0	0	1	7,500	5	1^st^
	P16	0	0	1	16,500	12	3^rd^
	TOTALS	4	5	7	106,700	99	-

* All registered adult patients; totals rounded to nearest 100 to preserve practice anonymity

**Table 2:** Recorded delivery of screening and brief alcohol intervention by practice

	Enhanced service status	Practice	All patients* (newly registered only)	FAST/ AUDIT-C screening test (%)	FAST/ AUDIT-C screening test (newly registered show patients only) (%)	Full AUDIT screening test (%)	Any form of alcohol intervention (%)
Area A	No enhanced service	P01	1400 (720)	1.09	2.08	0.00	0.00
		P02	16500 (1190)	0.01	0.17	0.35	0.44
		P03	7600 (330)	0.00	0.00	0.00	9.41
		P04	8000 (440)	0.00	0.00	0.00	9.89
		ALL	32100 (2680)	0.05	0.63	0.17	4.73
	DES only	P05	9800 (530)	0.00	0.00	0.31	5.63
		P06	3000 (180)	4.98	86.52	0.00	0.00
		P07	6800 (440)	0.01	0.23	0.00	0.01
		P08	600 (360)	56.21	92.29	5.20	7.89
		P09	8800 (880)	7.37	73.47	11.46	23.69
		ALL	29000 (2390)	3.92	47.54	3.68	9.23
Area B	DES + LES	P10	4900 (480)	7.06	72.18	0.00	23.82
		P11	1200 (350)	29.67	103.11	0.81	17.97
		P12	3100 (590)	15.51	80.00	0.73	9.45
		P13	4800 (350)	0.00	0.00	0.00	9.58
		P14	6200 (250)	4.14	104.02	0.00	0.37
		P15	7500 (500)	1.60	23.73	1.23	6.78
		P16	16500 (760)	0.10	2.11	8.50	6.14
		ALL	44200 (3280)	3.56	47.91	3.44	8.32

*All registered adult patients; totals rounded to nearest 100 [10] to preserve practice anonymity

#### Screening for an alcohol use disorder

Rates of short screening test administration (FAST or AUDIT-C) were lowest in practices with no enhanced service (0.05% (17/33539), 95% CI: 0.03-0.08). Rates were higher in practices signed up to either both the LES + DES (3.56% (1574/44202), 95% CI: 3.39-3.74) or the DES only (3.92% (1138/29065), 95% CI: 3.70-4.14). Taken as a proportion of newly registered patients only, rates were also lowest in non-incentivised practices (0.63% (17/2680), 95% CI: 0.40-1.01), compared to either practices signed up to the DES only (47.54% (1138/2394), 95% CI: 45.54-49.54) or those signed up to both the LES and DES (47.91% (1574/3285), 95% CI: 46.21-49.62).

For the full AUDIT, practices not signed up to an enhanced service had the lowest recorded rates of delivery (0.17% (57/33539), 95% CI: 0.13-0.22). Those signed up to both DES + LES services had slightly lower recorded rates (3.44% (1521/44202), 95% CI: 3.28-3.62) in comparison to practices signed up to only the DES (3.68% (1069/29065), 95% CI: 3.47-3.90).

#### Alcohol intervention

When all instances of recorded alcohol interventions were combined (brief advice, brief intervention and extended intervention), rates were highest in practices signed up to one or more enhanced service for alcohol. Combined intervention activity was highest in practices signed up to either the DES only (9.23% (2684/29065), 95% CI: 8.91-9.57) or both the DES + LES (8.32% (3677/44202), 95% CI: 8.06-8.58). Rates were lowest in practices not signed up to either enhanced service (4.73% (1585/33539), 95% CI: 4.50-4.96).

### Qualitative results

Ten of the 14 GP interviewees were based in practices that participated in the quantitative phase of the research. An equal representation of practices from each NHS area was achieved. Further participant details are provided in Table 3, with overarching interview themes, including where key differences emerged between GPs working in incentivised versus non-incentivised practices, described below.

**Table 3:** Summary characteristics of qualitative interview participants

		N [14]
Gender	Male	7
	Female	7
Experience in practice	>5 years	4
	5-15 years	3
	>15 years	7
Employment status	Partner	7
	Salaried GP	6
	Registrar	1
Location	Area A	7
	Area B	7
Enhanced service status	No Enhanced Service	3
	Directed Enhanced Service	4
	Directed Enhanced Service & Local Enhanced Service	7

#### The hierarchy of incentive schemes

All GPs across our sample prioritised the delivery and recording of QOF-related activity above that for other incentive schemes, and this hierarchy was reinforced via administrative and computing systems primarily designed to support the collection of QOF data. For GPs based in practices signed up to an alcohol enhanced service, as payment was based on the number of screening tests completed, the administration and recording of any subsequent preventative activity, such as the delivery of a brief intervention for alcohol, was less systematic. Moreover, screening activity was typically devolved to nurses and healthcare assistants as part of standard annual health checks or new patient registrations. GPs reported being unlikely to formally screen patients for heavy drinking during a consultation.

*“…you’re driven by what’s important or what you have to categorise. Somebody smoking is QOF-related so I find a way to Read Code somebody’s smoking status… So if you were saying would I record* ‘Delivered brief intervention on alcohol’*, I wouldn’t. Unless there was QOF driven reason for it, or it was important to put in their notes…”*

**GP11, male, DES + DES**

*“We have better systems in the practice to make sure that the QOF data is collected and there are more reminders on the screen if it’s not done. Back office staff will chase people up and things like that.”*

**GP2, male, DES**

#### Belief in the efficacy of screening and alcohol interventions

Irrespective of practice incentive status, most GPs believed that alcohol interventions could be effective in certain contexts, and with certain patients. However, there were also situations in which such an approach was viewed as unlikely to be impactful. Only one participant expressed an unreservedly positive view on their effectiveness. For some interviewees, this lack of faith in the effectiveness of brief alcohol interventions related to a belief that a patient needed to be ‘ready to change’ for interventions to work. As such, many GPs restricted their delivery (and in turn, recording) of alcohol interventions to patients that they judged as being ‘ready to change’.

*“I suppose one of the key things I feel with alcohol to some extent is, I suppose people have to be wanting to change before you can take them too far down the road of an intervention. And so sometimes yes they know they’re drinking too much but they’re not that ready to change, so going through a whole pathway doesn’t always help.”*

**GP2, male, DES**

*“You can feel like you’ve had a very good consultation and they’ll still go on drinking. Or you can do something really quickly and say ‘You know for heaven’s sake you’ve got to stop drinking.’ And they’ll come back in three months and say ‘Do you know when you said that I was really shocked, I’ve stopped drinking.’ And you think ‘Oh my goodness!’ It’s very hard to predict who you’re going to have an effect on.”*

**GP3, female, no enhanced service**

*“I’m realistic, it doesn’t work every time… that’s one of the mysteries, you don’t quite know who it’s gonna work with, or when it’s gonna work.”*

**GP7, male, DES + LES**

#### The challenge of coding complex and sensitive conditions

Given the critical role of Read Code data in evidencing performance for payment purposes, and supporting continuity of care, most GPs were anxious to code clinical activities correctly. However even in incentivised practices, there was low awareness of the appropriate Read Codes for screening and brief alcohol interventions, an issue potentially exacerbated by the existence of over 280 possible alcohol-related codes. [35] Further, many interviewees questioned the applicability of the available Read Code vocabulary to complex health conditions or behaviours that were less easily quantifiable, and thus codified. A few participants also highlighted the adverse consequences for patients potentially arising from formally recording their alcohol status, both in terms of financial impacts (e.g. higher health insurance costs), and the wider stigma associated with substance use disorders. When in doubt, some GPs reported using free text to record such issues rather than formal Read Coding. Indeed, irrespective of incentive scheme, it appeared that care delivery and care recording were not always tightly aligned.

*“Because, you know the situation is usually so complex, in terms of the person’s own personal and social history and what’s led them to heavy drinking…Read coding, simple coding can’t capture all that sort of, not by a long shot…you could perfectly well have instances in which the codes tell you that the doctor or the nurse has done the right things in terms of an intervention. But actually if the relationship and the trust and the understanding of the person’s social context isn’t there, then you don’t know the whole story.”*

**GP6, male, no enhanced service**

*“I always struggle to find any Read Codes for alcohol…I wouldn’t use specific Read Codes for an intervention delivered, I would record more what I say I did in free-text…I haven’t found so far any benefit from using codes…if you want to say a number of units a week then you’d really have tie a patient down…and somebody says oh I had three glasses of wine and a whiskey, tonight, somebody might classify that as four units but in reality it’s more nearer seven isn’t it? So if somebody was saying I drink that every night, seven times seven is 49, and then seven times four is 28, so you can say I see your alcohol consumption used to be 49 and now it’s 28, and it could just be the inaccuracies of data collection. So for me I don’t want to tie something down…because you could draw false inferences from data that isn’t that accurate.”*

**GP11, male, DES + LES**

#### The role of the GP within the patient-centred consultation

All GPs expressed an overriding concern to deliver a patient-centred consultation, informed by a thorough understanding of their wider social and familial circumstances. Whilst interviewees acknowledged the importance of recording treatment to support continuity of care, most saw Read Code data of limited value in terms of documenting a patient’s wider circumstances. This was seen as a particular weakness when it came to recording alcohol intervention delivery. In contrast, Read Codes were viewed as a more appropriate means of capturing the nurse-led activities that supported the GP role, particularly alcohol screening test administration.

*“I think often people in government, administrative bodies they say why aren’t we doing more about alcoholism in the UK forget that when patients come to the doctors it’s not a hole in the wall situation…doctors aren’t automatons and each patient is an individual.”*

**GP12, female, DES + LES**

*“I don’t like codes; you know…I’m a clinician, I love the clinical encounter… the commitment [is] to what has gone on with the patient.”*

**GP7, male, DES + LES**

*“As long as I know somebody’s drinking 50 units and I know that I’ve talked them through it and I know that they’re coming back to see me about it, whether I’ve coded it on the system or not, so what? The intervention’s been done.”*

**GP5, female, DES**

## Discussion

The introduction of financial incentives in England appears to have had some success in encouraging primary care providers to identify and support patients to reduce heavy alcohol consumption. However, whilst rates of screening and brief alcohol intervention delivery were higher in incentivised practices, this trend needs to be set against very low levels of recorded activity overall. Since the national DES incentivised the screening of newly registered patients only, a relatively small group within the wider patient population, such low rates may be expected. Our estimated rates of short screening test administration amongst newly registered patients indicate a more sizeable reach for the national enhanced service scheme. However it must be emphasised that these rates are estimates only: as the 100% + rates achieved by two of our practices indicate, alcohol screening tests were conducted with both new as well as existing registered patients.

Previous research by Purshouse et al suggests that if such a screening programme is implemented effectively, it would achieve around 40% coverage of the registered adult population over a ten year period, leading to reduced health service costs and increased health benefits [36]. However, this scenario is based on an assumption that a brief intervention for alcohol is delivered immediately after a patient screens positive for risky alcohol consumption. In many of our practices, there seemed to be a mismatch between screening patients for heavy drinking, and the delivery of subsequent advice or counselling about alcohol. Available prevalence data would suggest that approximately one in four patients [37] are likely to be drinking excessively, meaning that one might expect that at least a quarter of all patients screened would have received an alcohol intervention. In reality, for some practices, intervention delivery rates appeared much lower than expected. Yet, in others, the rates were much higher when compared with the proportion actually screened. We could not determine if this was due to an absence of coding or a lack of follow through to advice or counselling. We were not told of any deliberately erroneous or ‘over-coding’ by GPs incentivised for enhanced performance. However our interview findings suggest that additional factors beyond monetary incentives shaped routine practice. In particular, despite consistent evidence to the contrary, [5] many physicians remain unconvinced of the effectiveness of brief alcohol interventions: a barrier to delivery raised in previous implementation literature in this field [38].

The strength of our study was the mixed methods design, which helped provide a rich and nuanced understanding of the impact of financial incentives on GPs’ delivery and recording of screening and brief alcohol interventions. By using routinely recorded data as a measure of activity, we also provide a valuable insight into the rates of alcohol prevention work being delivered in real world as opposed to research trial settings.

However, there are several limitations to our data. Practices were not randomly sampled and thus there was a potential for self-selection bias. [39] In the absence of corroborating observational data (such as consultation recording), it was only possible to assess activity rates via formal coding. It is possible that these data were not an accurate record of all care provided, [40] particularly given our searches were limited to Read Coded rather free text data. Moreover, as we extracted aggregated counts of each variable of interest at practice level, there is potential for double-counting: that is an individual patient may have been screened twice with both shorter and longer tools; or received both brief advice as well as an extended intervention during the period of interest. Changes in the Read Codes used to record alcohol interventions since 2008 make it challenging to compare trends pre and post the introduction of financial incentives. Thus whilst our study suggests there are significant differences in delivery rates between incentivised and non-incentivised practices, these trends cannot be interpreted as causal.

The majority of interviewees were drawn from practices signed up to at least one enhanced service for alcohol. Interview accounts may have differed if we had recruited more ‘non-incentivised’ practitioners, but these were less inclined to be recruited. Further, whilst overall, recorded levels of alcohol prevention activity were significantly higher in incentivised practices, rates at individual practice level were nevertheless low even for some incentivised practices (P06, P07 and P16), suggesting that our sample captured a range of perspectives. In addition, despite the central role they appear to play in delivering alcohol screening tests, we did not interview nurses in this study. This study sampled 16 practices and interviewed 14 GPs across two NHS areas in one region in England, representing approximately eight per cent of the total practice population. In comparison to other parts of the UK, Northern England is relatively deprived [41] and has higher average alcohol consumption [42], potentially limiting the generalisability of our data. Whilst the low delivery rates reported here are unlikely to be higher elsewhere, a larger study would be required to validate our findings.

To some extent, our findings support previous research, suggesting that financial incentives can have positive impacts on screening and brief alcohol intervention delivery in primary care. [21, 43, 44] Interestingly, our results do not indicate that there was any cumulative effect for practices that were signed-up to both the local and national incentive schemes. Given that the GP interviews suggested that routine FAST or AUDIT-C delivery was more likely when embedded within nurse-delivered practice and associated recording systems (such as registering a new patient, as incentivised by the DES), limited instances of opportunistic screening (as incentivised by the LES) might be expected.

However, the recorded activity rates reported here were comparatively low for all outcomes of interest, irrespective of financial incentive status. Alongside the design of the scheme, this may be also due to the low level of remuneration associated with the national DES in particular. For example, practices participating in the QOF+ scheme assessed by Hamilton et al, were paid up to £5,607 for screening and delivering a brief intervention to all eligible patients. [45] DES practices in this study would have received just £2.38 for each newly registered patient screened, meaning they would need to screen around 2,300 patients per annum to achieve similar financial returns. Our qualitative findings suggest that differing incentive schemes are not seen as equal by GPs. Specifically, the QOF has the largest effect on practice income, so the delivery and recording of enhanced service activities are often accorded lower priority. [46]

At the same time, a strong theme from our interviews was the challenge that GPs experience when recording complex and potentially stigmatising conditions such as harmful alcohol consumption. In particular, their concern to preserve ‘patient-centred’ consultations sometimes clashed with the need to record simple diagnoses and outcomes, especially when uncertainty or sensitivity was at play. [47] This tension has been described as a *“rational-reality gap”,* [48] requiring clinicians to maintain a *“dual orientation”* towards coding. [49] Whilst other research suggests that financial incentives such as QOF serve to promote an increasingly biomedical agenda in terms of the management of chronic and complex health issues in primary care [50], our results suggest more nuanced behaviour on the part of GPs. Importantly, we found numerous examples of GPs adapting the system to allow their behaviour to more closely align with their preferred ‘patient-centred’ approach, with the ‘tick-box’ elements of alcohol-related care devolved to nurses.

## Conclusions

This study provides further evidence that policy initiatives that focus solely on the extrinsic motivations of GPs, such as financial incentives, are unlikely to have the desired level of impact without acknowledging the values, attitudes and beliefs that also shape care. The incongruity we observed between recorded rates of screening versus actual alcohol intervention delivery also highlight the potentially distorting effects of pay-for-performance on healthcare recording, and in particular, of incentivising process as opposed to outcomes [51]. Our findings also suggest that some incentive schemes are more impactful than others, with QOF unarguably most influential as far as English primary care is concerned. Given the radical reduction to the number of clinical indicators covered by the current QOF, [52] the addition of screening and brief alcohol intervention in the future seems unlikely. This is despite that the fact that over one in four patients continue to drink above recommended levels, [37] whilst three quarters of the English population do not have any of the diseases listed in the QOF. [53] Since April 2015, the national enhanced service for alcohol has been withdrawn, although local level incentive schemes remain in place for some areas [54], and there is now a contractual requirement for practices to identify newly registered adult patients drinking above recommended levels [55]. Based on the findings from this study, which highlight the substantial challenges experienced by GPs seeking to prioritise non-incentivised care over their routine management of QOF conditions, this seems a risky strategy. If NHS England is to deliver on its promise of “hard-hitting” action on tackling risky lifestyle behaviours in the future, [56] a fresh look at the financing and organisation of preventative care is urgently required.

## Additional file

Additional file 1: Interview guide for General Practitioners. (DOCX 19 kb)

## Abbreviations

AUDIT: Alcohol Use Disorders Identification Test
AUDIT C: Alcohol Use Disorders Identification Test Consumption
DES: Directed Enhanced Service
FAST: Fast Alcohol Screening Test
LES: Local Enhanced Service
NHS: National Health Service
QOF: Quality and Outcomes Framework
