# Supplementary material for: Impact of financial incentives on alcohol intervention delivery in primary care: a mixed-methods study
Source: BMC Fam Pract. 2016 Nov 25;17:165. doi: 10.1186/s12875-016-0561-5 (PMC5124277; doi:10.1186/s12875-016-0561-5)
Supplement: Additional file 1: — Interview guide for General Practitioners. (DOCX 19 kb) [file 12875_2016_561_MOESM1_ESM.docx]

**Interview guide for General Practitioners**

### Section 1: Background, roles and responsibilities

1. Could you tell me a bit about this practice?
2. What is your role within this practice?

### Section 2: Delivering screening and brief interventions (SBI) for alcohol

1. How long have you been involved in delivering alcohol SBIs?
2. Could you describe the process of delivering alcohol SBIs here at this practice?
3. How would you describe SBIs in terms of ease of delivery?
4. Do you think alcohol SBIs work?:
   - What evidence do you have?
5. Do you have experience of using Read Codes to record SBIs for alcohol?
6. What drives you to record SBIs using Read Codes?
7. Are there any aspects of alcohol SBIs that you find difficult to record?

### Section 3: Using GP Read Codes

1. Do you use Read Codes to record most aspects of patient consultations?
   - What do / don’t you record?
   - When might you prefer to use free text?
2. Do you find it easy to incorporate the use of Read Codes into the consultation process?
3. Is it straightforward to locate the correct Read Code in consultations?
   - Are some symptoms / types of treatment and care more difficult to code than others?
4. What support or guidance have you received on using Read Codes?
5. How important do you feel it is to use accurate and comprehensive Read Codes to record patient care?
